# M1 and M2 macrophages derived from THP-1 cells differentially modulate the response of cancer cells to etoposide

**DOI:** 10.1186/s12885-015-1546-9

**Published:** 2015-08-08

**Authors:** Marie Genin, Francois Clement, Antoine Fattaccioli, Martine Raes, Carine Michiels

**Affiliations:** 1 URBC, NARILIS, University of Namur, Namur, Belgium; 2 Laboratory of Biochemistry and Cellular Biology, NARILIS, University of Namur, 61 rue de Bruxelles, 5000 Namur, Belgium

**Keywords:** THP-1, Macrophage polarization, Cancer cells, Co-culture, Apoptosis

## Abstract

**Background:**

Tumor associated macrophages (TAMs) are present in high density in solid tumors. TAMs share many characteristics with alternatively activated macrophages, also called M2. They have been shown to favor tumor development and a role in chemoresistance has also been suggested. Here, we investigated the effects of M2 in comparison to M1 macrophages on cancer cell sensitivity to etoposide.

**Methods:**

We set up a model of macrophage polarization, starting from THP-1 monocytes differentiated into macrophages using PMA (Phorbol 12-myristate 13-acetate). Once differentiated (M0 macrophages), they were incubated with IL-4 and IL-13 in order to obtain M2 polarized macrophages or with IFN-gamma and LPS for classical macrophage activation (M1). To mimic the communication between cancer cells and TAMs, M0, M1 or M2 macrophages and HepG2 or A549 cancer cells were co-cultured during respectively 16 (HepG2) or 24 (A549) hours, before etoposide exposure for 24 (HepG2) or 16 (A549) hours. After the incubation, the impact of etoposide on macrophage polarization was studied and cancer cell apoptosis was assessed by western-blot for cleaved caspase-3 and cleaved PARP-1 protein, caspase activity assay and FACS analysis of Annexin V and PI staining.

**Results:**

mRNA and protein expression of M1 and M2 markers confirmed the polarization of THP-1-derived macrophages, which provide a new, easy and well-characterized model of polarized human macrophages. Etoposide-induced cancer cell apoptosis was markedly reduced in the presence of THP-1 M2 macrophages, while apoptosis was increased in cells co-cultured with M1 macrophages. On the other hand, etoposide did not influence M1 or M2 polarization.

**Conclusions:**

These results evidence for the first time a clear protective effect of M2 on the contrary to M1 macrophages on etoposide-induced cancer cell apoptosis.

## Background

Macrophages constitute a heterogeneous population of myeloid cells of the innate immune system involved in several processes in physiological as well as in pathological conditions. They are particularly active in inflammation and infection. Under such conditions, blood monocytes are recruited into the tissue where they differentiate into macrophages [1]. Macrophages display a high plasticity, which allows them to adapt their phenotype in response to different environmental stimuli [2]. Two major polarization states have been described for macrophages, the classically activated type 1 (M1) and the alternatively activated type 2 (M2). In 2002, Mantovani et al. [3] described these two macrophage phenotypes as extremes of a continuum of functional states. Classical activation of macrophages occurs following injury or infection. Macrophages are classically activated in vitro using bacterial cell wall components (such as LPS) and IFN-γ or TNF-α. M1 macrophages are characterized by the production of pro-inflammatory cytokines like TNF-α, IL-1β, IL-6 and IL-12. They also produce high levels of reactive oxygen and nitrogen species [4]. However, the expression of iNOS, the enzyme responsible for NO production by M1 macrophages, is specific to murine macrophages and is absent in human macrophages [5]. M2 macrophage polarization can be induced by different stimuli: IL-4 and/or IL-13, immune complexes and toll-like receptor, IL-1 receptor ligands or IL-10 [6]. Alternatively activated macrophages polarized by IL-4 and IL-13 are characterized by a limited production of pro-inflammatory cytokines, but they secrete anti-inflammatory cytokines like IL-10, CCL18 and CCL22. They are also characterized by the expression of several receptors like the mannose receptor CD206 (or MRC1), the scavenging receptor CD163, dectin-1 and DC-SIGN (Dendritic cell-specific intercellular adhesion molecule-3-grabbing non-integrin) [7, 8].

Solid tumors comprise not only malignant cells but also stromal host cells such as adipocytes, fibroblasts and hematopoietic cells, which are recruited from the blood vessels. Among these tumor infiltrated immune cells, macrophages are the most abundant, called tumor associated macrophages (TAMs) [9]. Many studies have shown that in malignant tumors, macrophages predominantly exhibit a M2-like phenotype [3]. M2 macrophages, on the contrary to M1 cells that are pro-inflammatory and cytotoxic, are immunosuppressive and favor angiogenesis and tissue repair [10]. Many studies have shown that tumor associated M2 macrophages improve tumor cell growth and survival and stimulate angiogenesis and metastases. In 2011, Shree et al. showed that cathepsin-expressing macrophages protect breast cancer cells from cell death induced by several chemotherapeutic drugs like taxol or etoposide [11]. Very recently, Mantovani and Allavena published a review summarizing the actual knowledge on the effect of anticancer therapies on TAMs [12]. However, a better understanding of this chemoprotective effect is still needed in order to design more efficient therapeutic strategies.

In order to study how macrophages could modulate tumor cells and in particular the tumor cell response to chemotherapeutic agents, we first set up a new and convenient model of human macrophage polarization. Macrophages were differentiated starting from the human monocytic cell line THP-1. Once differentiated in the presence of PMA, they can be polarized into M1 or M2 macrophages that express markers similarly to polarized macrophages obtained from freshly isolated monocytes. When HepG2 hepatoma cells or A549 lung adenoma cells were co-cultured with THP-1 M1 or M2 macrophages, they responded differentially to etoposide. In the presence of THP-1 M1 macrophages, the apoptosis of cancer cells induced by etoposide increased. On the opposite, M2 THP-1 macrophages were protective. This is the first demonstration that THP-1 polarized macrophages display functions similar to the ones described for polarized TAMs.

## Methods

### Cell culture

Human monocytic THP-1 cells were maintained in culture in Roswell Park Memorial Institute medium (RPMI 1640, Invitrogen) culture medium containing 10 % of heat inactivated fetal bovine serum (Invitrogen) and supplemented with 10 mM Hepes (Gibco, #15630-056), 1 mM pyruvate (Gibco, #11360-039), 2.5 g/l D-glucose (Merck) and 50 pM ß-mercaptoethanol (Gibco; 31350–010). THP-1 monocytes are differentiated into macrophages by 24 h incubation with 150 nM phorbol 12-myristate 13-acetate (PMA, Sigma, P8139) followed by 24 h incubation in RPMI medium. Macrophages were polarized in M1 macrophages by incubation with 20 ng/ml of IFN-γ (R&D system, #285-IF) and 10 pg/ml of LPS (Sigma, #8630). Macrophage M2 polarization was obtained by incubation with 20 ng/ml of interleukin 4 (R&D Systems, #204-IL) and 20 ng/ml of interleukin 13 (R&D Systems, #213-ILB). HepG2 and A549 cells were respectively cultivated in Dulbecco’s modified Eagle's minimal essential medium (DMEM medium 1 g glucose/l) (Gibco) and Minimum Essential Medium Eagle medium (MEM) (Gibco), both containing 10 % fetal bovine serum. In the co-culture experiments, THP-1 monocytes were differentiated in 6 Transwell inserts (membrane pore size of 0.4 μm, Corning, #3450). Macrophages and HepG2 cells were co-cultured in CO_2_ independent medium supplemented with 0.5 mM L-glutamine (Sigma, # G3126) and 3.75 g/l of D-glucose (Sigma, #50-99-7) for 16 h before being incubated with or without 50 μM etoposide (Sigma, #E1383) for 24 h. Macrophages and A549 cells were co-cultured in CO_2_ independent medium supplemented with 0.5 mM L-glutamine and 2.5 g/l of D-glucose for 24 h before being incubated with or without 50 μM etoposide for 16 h. In the monoculture experiments, 0.8 x 10^6^ THP-1 monocytes were differentiated and polarized in 6 well plates. Next, they were incubated in CO_2_ independent medium supplemented with 0.5 mM L-glutamine (Sigma, # G3126) and 3.75 g/l of D-glucose (Sigma, #50-99-7) for 16 h before being incubated with or without 50 μM etoposide (Sigma, #E1383) for 24 h.

### Immunofluorescence labeling and confocal microscopy

THP-1 monocytes were seeded at 100 000 cells/well in 24-well plates containing a coverslip and were differentiated as described here above. Undifferentiated monocytes were attached on coverslips by drying a PBS drop containing 100 000 cells. For labeling, cells were fixed for 10 min with paraformaldehyde 4 % in cold PBS, washed three times with 2 % PBS–BSA (bovine serum albumin) and incubated overnight at 4 °C with the primary antibody 1:100 diluted in 2 % PBS-BSA: anti-CD68 (KP1) from Abcam (ab955), anti-CD71 (H300) from Santa Cruz (sc-9099), anti-CD36 (H300) from Santa Cruz (sc-9154), anti-CD14 (1H5D8) from Abcam (ab181470). Cells were washed three times with 2 % PBS–BSA and then incubated for 1h with the secondary antibody. Alexa Fluor-488-conjugated anti-rabbit IgG antibody (Molecular Probes, #A11034) was used at 1/1000 dilution. Cells were then washed three times with PBS, the coverslips were mounted in Mowiol (Sigma) and observed with a confocal microscope (SP5, Leica).

### Cell viability (MTT assay)

THP-1 monocytes were seeded at 180 000 cells/well in 24 well plates and differentiated in macrophages as described. After incubation with IFN-γ ± LPS, cells were incubated 2 h with 500 μl of MTT reagent (2.5 mg/ml of PBS, Sigma #M2128) in the CO_2_ incubator. The media were then removed and 1 ml of lysis buffer (SDS 30 %/N,N-dimethyl-formamide 2:1 pH 4.7) was added per well. Plates were incubated at 37 °C and gently shaked at 70 rpm for 1 h. The absorbance was then measured at 570 nm.

### RT-qPCR

After the incubation, total RNA was extracted using the RNeasy mini kit and DNase protocol (Qiagen, #74104). mRNA contained in 2 μg total RNA was reverse transcribed using Transcriptor first strand cDNA synthesis kit (Roche, #4379012001). Amplification reaction assays contained SYBRGreen PCR Master Mix (Applied Biosystem, #4309155) and primers (IDT, 300 nM). RPS9 (40S ribosomal protein S9) was used as the reference gene for normalization and mRNA abundance was quantified using the threshold cycle method.

### ELISA

Cytokine secretion in the culture medium was assayed using an ELISA kit according to the procedure recommended by the supplier (CXCL10 (R&D System, #DIP100), IL-6 (R&D System, D6050), IL-10 (R&D System, D1000B), CCL18 (Abnova, #KA1757)).

Results are expressed in pg of cytokine normalized per μg of proteins assayed by the Pierce method after cell lysis using 60 μl of mammalian protein extraction reagent (78501 from Thermo Scientific).

### Analysis of CD206 plasma membrane expression by flow cytometry

THP-1 monocytes were seeded in T25 flask at 2.5 × 10^6^ cells/T25 and differentiated with PMA. After incubation with or without IL-4 and IL-13, cells were washed with cold PBS and detached with EDTA 5 mM. Cold PBS containing 5 % human heat inactivated serum (his) and 0.1 % NaN_3_ was added in the flasks and the cell suspension put in FACS tubes. Cells were counted and 0.5 × 10^6^ cells were resuspended in a total of 1 ml of PBS 5 % his 0.1 % NaN_3_. Cell suspension was centrifuged 5 min at 200 g and 4 °C and the pellet resuspended with 1 ml of PBS 5 % his 0.1 % NaN_3_. This washing step was performed twice. The pellet was next resuspended with human truStain FcX (BioLegend, #422301) diluted 20 x in PBS. A total volume of 50 μl was used for resuspension. The suspension was then incubated at room temperature for 10 min. Cells were centrifuged 5 min at 200 g and 4 °C. The pellet was resuspended with 50 μl of primary anti-CD206 antibody diluted 5 times in PBS 5 % his 0.1 % NaN_3_ and incubated 30 min at 4 °C. Cells were also incubated with the control isotype corresponding to each primary antibody. Primary antibodies are PE (Phycoerythrin) mouse anti-human CD206 (BD Pharmingen, #555954) and PE mouse IgG1 κ isotype control (BD Pharmingen #555749). After incubation, PBS 5 % his 0.1 % NaN_3_ was added and the suspension centrifuged 5 min at 200 g and 4 °C. Three washes with PBS 5 % his 0.1 % NaN_3_ were next performed. The pellet was resuspended with 2 % paraformaldehyde (in cold PBS) and incubated 20 min at 4 °C. Suspension was centrifuged 5 min at 200 g 4 °C and the pellet resuspended with glycine 0.1 M (in cold PBS) and incubated 10 min at 4 °C. A last centrifugation of 5 min at 200 g and 4 °C was performed before cell resuspension in 1 ml of PBS 5 % his 0.1 % NaN_3_. Cells were analyzed by flow cytometry with a FACScalibur (BD Biosciences).

### Western blotting

Cells were seeded in 6 well plates (Costar; 250000 HepG2 cells/well and 125000 A549 cells/well) 1 day before incubation with macrophages. After the incubation, proteins were extracted and PARP-1 and caspase-3 protein abundance was assessed by western blotting as described previously [13]. Primary antibodies are rabbit anti-caspase-3 antibody (Cell Signaling, #9662) and mouse anti-PARP1 antibody (BD Pharmingen, #551025). Primary antibodies mouse anti-*β*-actin (Sigma, #A5441) or mouse anti-α-tubulin (Sigma, # T5168) were used for normalization. IRDye 800CW-conjugated goat anti-rabbit antibody (H + L; Licor, #926-32211), IRDye 800CW-conjugated goat anti-mouse antibody (H + L; Licor, #926-32210) and IRDye 680LT-conjugated goat anti-mouse antibody (H + L; Licor, # 926–68020) were used as secondary antibodies. Quantitative analysis of fluorescence intensity was measured using the Odyssey Classic Infrared Imaging System (Licor).

### Caspase activity assay

The fluorogenic substrate Ac-DEVD-AFC was used to measure caspase-3 and caspase-7 activity according to Lozano *et al.* [14]. Cell extracts were prepared as described by Wellington *et al.* [15]. HepG2 or A549 cells were seeded in 6 well plates (Costar; 250000 HepG2 cells/well- 125000 A549 cells/well) 1 day before incubation with macrophages. After the incubation, proteins were extracted and caspase activity was measured in the different samples as described previously [13].

### Flow cytometry analysis of Annexin V/Propidium iodide staining

HepG2 or A549 cells were seeded in 6 well plates (Costar; 250000 HepG2 cells/well- 125000 A549 cells/well) 1 day before incubation with macrophages. After the incubation, tumor cells were harvested with trypsin/EDTA and stained using FITC Annexin V apoptosis Detection Kit I (BD Pharmingen #556547). Results were analyzed by flow cytometry (FACSCalibur, BD).

### Statistical analysis

Statistical analyses were performed using the Sigma Stat software. For some analyses, values did not follow a Gaussian distribution. In order to deal with this absence of normality, statistical analyses were performed on log-transformed data. In order to facilitate interpretation, untransformed data are shown.

## Results and discussion

### Monocyte differentiation into macrophages

**Fig. 1:**
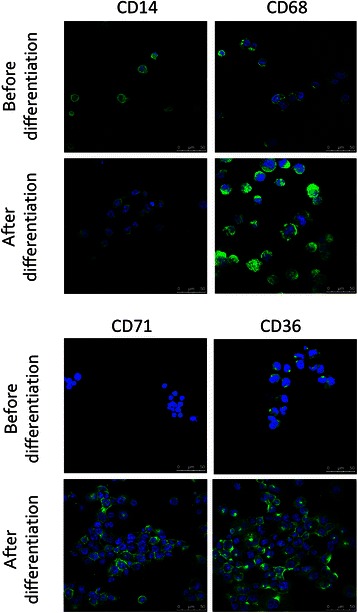
THP-1 monocyte differentiation in macrophages. THP-1 cells were incubated 24 h in the presence of 150 nM PMA and then in RPMI medium during 24 h. Cells were then fixed and immunolabeled for CD14, CD68, CD71 or CD36 using specific antibodies (green). Nuclei were detected with To-pro3 (blue)

Human THP-1 monocytes were differentiated into macrophages by an incubation in the presence of phorbol 12-myristate 13-acetate (PMA). Different PMA concentrations and incubation times were tested (data not shown). A 24 h incubation in the presence of 150 nM PMA followed by 24 h in control medium was finally selected as differentiation protocol. Cells became adherent and the expression of recognized macrophage markers, CD68 (cluster of differentiation 68) [16], CD71 [17] and CD36 [18], analyzed by immunofluorescence staining to confirm the monocyte-to-macrophage differentiation, also clearly increased. The expression of CD14, which decreases with macrophage differentiation [19], was also studied and confirmed the differentiation (Fig. 1).

### THP-1 polarization into pro-inflammatory M1 macrophages

The classical protocol for M1 polarization is to incubate macrophages in the presence of IFN-γ alone or in combination with LPS [6], in general for 24 h. While IFN-γ is used at 20 ng/ml in most studies the LPS concentration varied from 10 ng to 1 μg/ml according to the reports [20, 21].

**Fig. 2:**
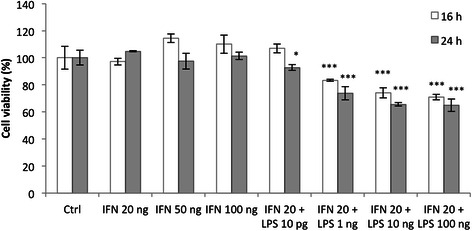
Effects of IFN-γ and/or LPS on THP-1 macrophage viability after 16 or 24 h of incubation. THP-1 macrophages were incubated in RPMI medium with IFN-γ (20, 50 or 100 ng/ml) alone or in combination with LPS at different concentrations. After 16 and 24 h of incubation, cell viability was measured by a MTT assay. Results are expressed as means ± 1 S.D. (*n* = 3). Statistical analysis was carried out with a two-way ANOVA test followed by a Holm-Sidak post-test. * or ***: significantly different from the corresponding control (Ctrl) respectively with p < 0.05 or 0.001

Based on the literature, we tested different concentrations of LPS, varying from 1 to 100 ng/ml, combined with 20 ng/ml of IFN-γ and we incubated THP-1 macrophages during 16 or 24 h. We observed a high cytotoxicity, which increased with the LPS concentration: cell viability, measured by a MTT assay, decreased from 100 % in control cells to 65 % after 24 h incubation with 10 ng/ml of LPS + 20 ng/ml of IFN-γ. No toxicity was observed with IFN-γ alone (Fig. 2). The cytotoxicity induced by LPS on macrophages has been already described [22, 23]. To reduce the LPS induced cytotoxicity, Hirose and colleagues worked with lower LPS concentrations and incubated macrophages for M1 polarization with 10 pg/ml of LPS + 20 ng/ml INF-γ for 18 h [22]. We thus incubated M0 THP-1 macrophages during 16 or 24 h with 10 pg/ml LPS + 20 ng/ml IFN-γ. In these conditions, the cell viability was not affected after 16 h incubation and only slightly (93 % cell viability) after 24 h incubation (Fig. 2).

**Fig. 3:**
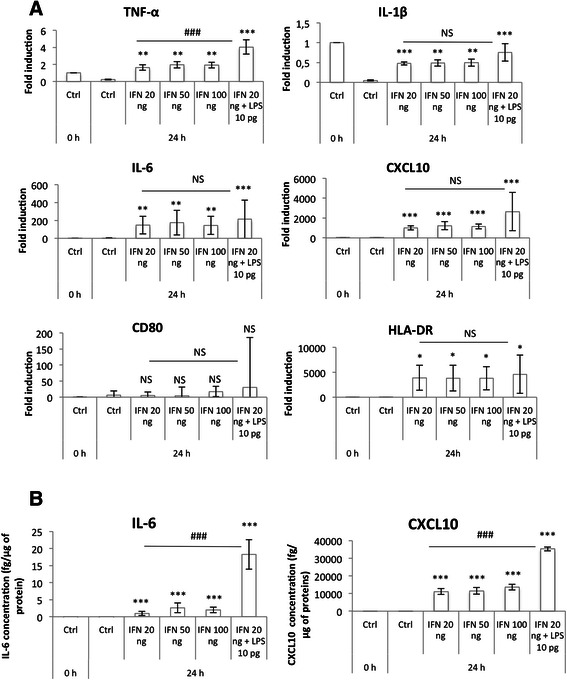
M1 macrophage marker expression. THP-1 macrophages were incubated with IFN-γ (20, 50, 100 ng) and/or LPS 10 pg/ml during 24 h. (**a**) mRNA expression of M1 macrophage markers was studied by RT-qPCR and normalized by RPS9 expression. Results are expressed as means ± 1 S.D. (*n* = 3). (**b**) IL-6 and CXCL10 secretion in culture medium was measured by ELISA. Results are expressed as means ± 1 S.D. (*n* = 3). Statistical analysis was carried out with a one-way ANOVA test followed by a Holm-Sidak post-test. NS: not significantly different. *, ** or ***: significantly different from the corresponding control (Ctrl) respectively with p < 0.05, 0.01 or 0.001; ###: significantly different with p < 0.001. Statistical analyses were performed on non-transformed data for TNF-α (**a**), IL-1ß (**a**), IL-6 (**b**) and CXCL10 (**b**) and on log-transformed data for IL-6 (**a**), CXCL10 (**a**), CD80 (**a**) and HLD-DR (**a**)

Macrophage M1 polarization was then assessed by measuring the expression of several classical M1 markers: TNF-α, IL-1β, IL-6 and CXCL10, which are pro-inflammatory cytokines, and CD80 and HLA-DR, two membrane receptors, both at the mRNA level using RT-qPCR (Fig. 3a) and at the protein level by ELISA (for IL-6 and CXCL10) (Fig. 3b). An increased pro-inflammatory marker expression profile was obtained by incubation with IFN-γ combined with 10 pg/ml of LPS in comparison to IFN-γ alone. TNF-α and IL-1β were expressed in control M0 macrophages, but their expression decreased after 24 h in control medium. This could be due to PMA used for monocyte-to-macrophage differentiation, which has been described to up-regulate their expression [24]. On the other hand, their expression was highly increased in macrophages incubated in the presence of LPS and IFN-γ.

**Fig. 4:**
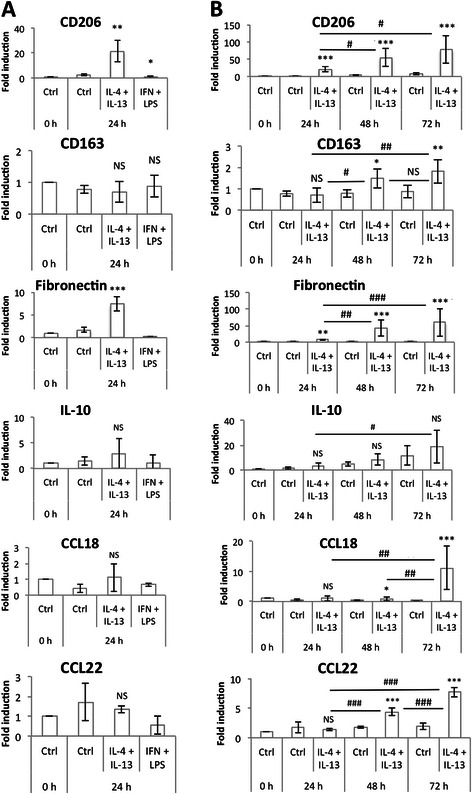
M2 macrophage marker mRNA expression. (**a**) THP-1 macrophages were incubated during 24 h either with IL-4 and IL-13 (20 ng/ml each) or with IFN-γ 20 ng/ml and LPS 10 pg/ml and mRNA expression of M2 macrophage markers was studied by RT-qPCR and normalized by RPS9 expression. Results are expressed as means ± 1 S.D. (*n* = 3). (**b**) THP-1 macrophages were incubated with IL-4 and IL-13 (20 ng/ml each) during 24, 48 or 72 h and mRNA expression of M2 macrophage markers was studied by RT-qPCR and normalized by RPS9 expression. Results are expressed as means ± 1 S.D. (*n* = 3). Statistical analysis was carried out with a one-way ANOVA for figure A and a two-way ANOVA for figure B, followed by a Holm-Sidak post-test. NS: not significantly different. *, ** or ***: significantly different from the corresponding control (Ctrl) respectively with p < 0.05, 0.01 or 0.001; ###: significantly different with p < 0.001. Statistical analyses were performed on log-transformed data for CD206 (**b**), fibronectin (**b**) and CCL18 (**b**)

We also checked the expression at the mRNA level of several M2 markers (CD206, CD163, fibronectin, IL-10, CCL18 and CCL22) in M1 macrophages, but in our conditions, we observed no significant expression of these genes (Fig. 4a). This was not the case when higher LPS concentrations were used for macrophage polarization. Indeed, after 24 h of incubation with 10 ng/ml of LPS + 20 ng/ml IFN-γ, the mRNA abundance of CCL18 increased (data not shown). CCL18 has been frequently described as a M2 macrophage marker, induced by IL-4, IL-13 and IL-10 [6, 25]. In 2013, Chanput *et al.* [26] published a model of THP-1 macrophage polarization in M1 and M2 macrophages. To polarize macrophages in M1 cells, they incubated cells with 20 ng/ml IFN-γ plus 1 μg/ml LPS. In these conditions, they measured higher levels of expression for several M2 macrophage markers (IL-10, CCL17, CCL18) in M1 macrophages than in M2 (polarized after 24 h incubation with 20 ng/ml IL-4). This result confirms our hypothesis that the incubation of THP-1 macrophages with high LPS concentrations might induce an unspecific expression of M2 macrophage markers in pro-inflammatory macrophages.

In conclusion, incubation of THP-1 macrophages with IFN-γ 20 ng/ml and LPS 10 pg/ml during 24 h induces their polarization into M1 macrophages.

### THP-1 polarization into anti-inflammatory M2 macrophages

Macrophage polarization into alternatively activated macrophages, also called M2 cells, is induced *in vivo* and *in vitro* by IL-4 and IL-13 stimulation [6]. In most of the studies in which murine or human primary macrophages were polarized into M2 macrophages, incubations of 16 or 24 h with 20 ng/ml of IL-4 alone or combined with 20 ng/ml of IL-13 have been generally used [20, 22, 23].

**Fig. 5:**
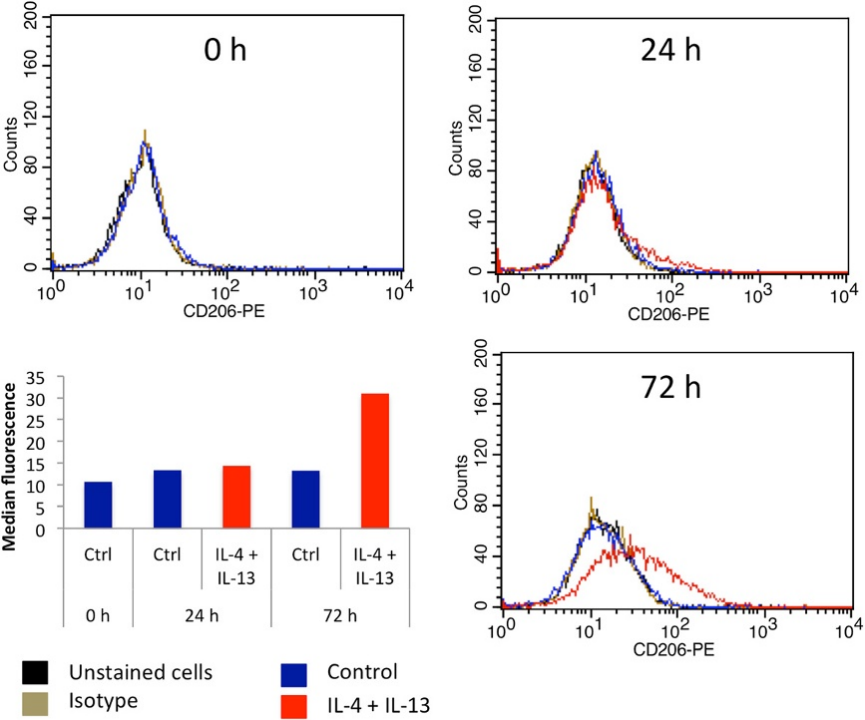
Cell surface CD206 expression by M2 macrophages. THP-1 macrophages were incubated during 24 or 72 h with control medium with or without IL-4 and IL-13. CD206 protein expression on macrophages was analyzed by flow cytometry with a specific antibody coupled to PE. Two controls were performed: some cells were unstained and others stained with a control isotype. The graph presents the histogram median of one of each sample

**Fig. 6:**
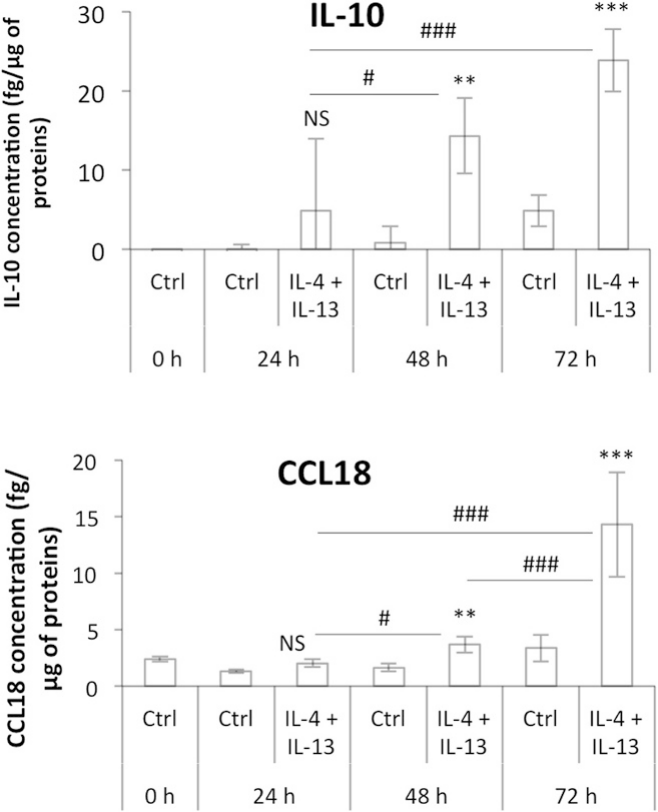
M2 macrophage secretion of IL-10 and CCL18. The IL-10 and CCL18 secretion in culture medium by macrophages was measured by ELISA. Results are expressed as means ± 1 S.D. (*n* = 3). Statistical analysis was carried out with a two-way ANOVA test, followed by a Holm-Sidak post-test. NS: not significantly different. ** or ***: significantly different from the corresponding control (Ctrl) respectively with p < 0.01 or 0.001; # or ###: significantly different respectively with p < 0.05 or 0.001. Statistical analyses were performed on log-transformed data for CCL18

We incubated M0 THP-1 macrophages with IL-4 and IL-13 at a concentration of 20 ng/ml during 24, 48 or 72 h. The M2 phenotype was characterized by studying the mRNA and protein abundance of several M2 markers: CD206, CD163, fibronectin, IL-10, CCL18 and CCL22. After 24 h incubation, the expression of CD206, fibronectin and IL-10 was slightly increased whereas CD163, CCL18 and CCL22 expression was unchanged. If the incubation time with IL-4 and IL-13 was increased to 48 and even further to 72 h, the mRNA abundance of all M2 markers was much higher (Fig. 4b). The expression pattern of CD206, IL-10 and CCL18 was confirmed at the protein level by FACS analysis for CD206 (Fig. 5) and by ELISA for IL-10 and CCL18 (Fig. 6). No expression of any M1 macrophage marker was evidenced in M2 polarized macrophage after 72 h incubation with IL-4 and IL-13 (data not shown).

When compared to results obtained with primary macrophages differentiated from blood-isolated monocytes, the polarization of macrophages derived from THP-1 seems to require a longer incubation time (data not shown, [20]). Indeed, Martinez and colleagues measured a high CCL18 mRNA expression in primary macrophages incubated 16 h with 20 ng/ml of IL-4 (M1:M2 ratio of −19) while 72 h of incubation with IL-4 and IL-13 were required to induce CCL18 expression and to detect a secretion of CCL18 in the culture media of THP-1-derived M2 macrophages.

### Effect of M1 and M2 macrophages on cancer cell apoptosis

In order to study the effects of M1 (pro-inflammatory and anti-tumoral) and M2 (anti-inflammatory and pro-tumoral) THP-1 macrophages on cancer cell response to a chemotherapeutic agent, each cell population was co-cultured with HepG2 (human hepatoma) cells in indirect contact using Transwell inserts. Monocytes were seeded on inserts made of a membrane with 0.4 μm pores, which allowed the exchange of soluble factors but not the trans-migration of cells. THP-1 monocyte differentiation was launched at different days for M2, M1 and M0 macrophages in order to obtain differentiated and polarized macrophages on the same day. 250,000 HepG2 cells were seeded in 6 well plates 24 h before the end of macrophage polarization. This cell density was chosen in order to have a 1:1 ratio between tumor cells and macrophages co-cultured in serum free medium. Serum free medium was used because serum protects HepG2 cells against apoptosis induced by etoposide (data not shown). After 16 h of co-culture, the two cell populations were incubated in the presence of 50 μM of etoposide added directly into the wells. Cells were further incubated with etoposide for 24 h.

**Fig. 7:**
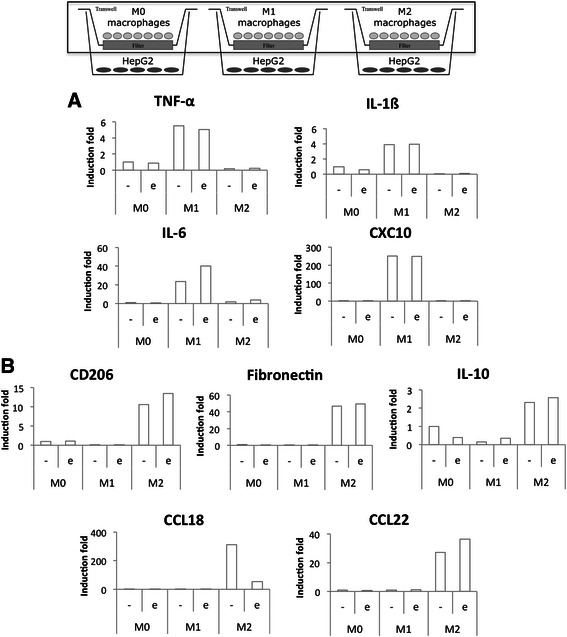
Study of M1 (**a**) and M2 (**b**) polarization marker mRNA expression in co-cultured macrophages. Macrophages were co-cultured in indirect contact with HepG2 cells during 16 h before incubation with or without 50 μM etoposide (+/− e) during 24 h. After the incubation, macrophage RNA was extracted, retro-transcribed and the mRNA expression of M1 and M2 macrophage markers was studied by RT-qPCR (*n* = 1)

**Fig. 8:**
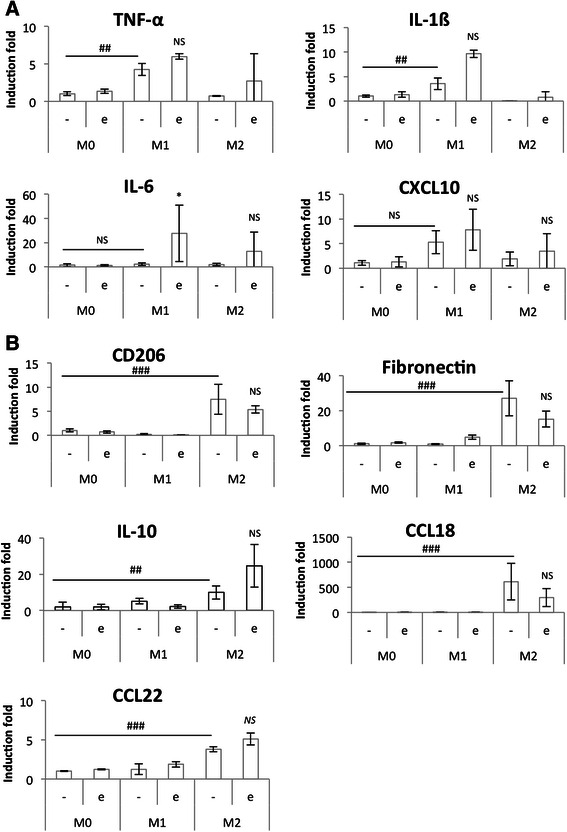
Study of M1 (**a**) and M2 (**b**) polarization markers mRNA expression in etoposide-incubated macrophages. THP-1 macrophages were differentiated (M0) and polarized in M1 and M2 macrophages by respectively 24 h with IFN-γ (20 ng/ml) + LPS (10 pg/ml) (M1) and 72 h with IL-4 and IL-13 (20 ng/ml each- M2)). Once polarized, they were incubated in CO_2_-independent medium (+3,75 g D-glucose/l) during 16 h before incubation with or without 50 μM etoposide (+/− e) during 24 h. After the incubation, mRNA expression of M1 and M2 macrophage markers was studied by RT-qPCR and normalized by RPS9 expression. Results are expressed as means ± 1 S.D. (*n* = 3). Statistical analysis was carried out with the two-way ANOVA test followed by a Holm-Sidak post-test NS: not significantly different. *: significantly different from the corresponding control with p < 0.05; ## or ###: significantly different respectively with p < 0.01 or 0.001. Statistical analyses were performed on log-transformed data for all the genes

At the end of the incubation, RNA was extracted from macrophages and RT-qPCR was used to measure M1 and M2 macrophage marker expression (Fig. 7). Incubation in CO_2_ independent medium with or without etoposide had no effect on macrophage polarity. Indeed, IL-6 was the only M1 marker clearly affected by the presence of etoposide. Regarding M2 macrophage markers, only CCL18 expression was strongly reduced in cells incubated with etoposide. The same experiment was performed on monocultures of macrophages incubated in the same conditions and the M1 and M2 marker expression was similar to the one measured in co-cultures (Fig. 8). Incubation of pro-inflammatory M1 macrophages in the presence of etoposide not significantly increased IL-6 and IL-1ß mRNA expression. This increase is probably due to p38 MAPK activation by etoposide [5]. Once activated, p38 MAPK induces TNF-α, IL-ß and IL-6 expression. Moreover, IL-6 and CXCL10 levels were higher in M1 macrophages in co-culture with HepG2 cells than in monoculture.

**Fig. 9:**
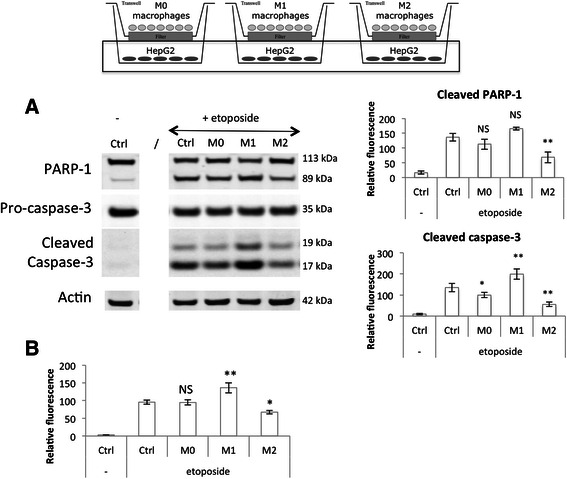
Modulation of HepG2 cell apoptosis induced by etoposide by co-cultured M0, M1 and M2 macrophages. Macrophages were co-cultured in indirect contact with HepG2 cells during 16 h before incubation with or without 50 μM etoposide (+/− e) during 24 h. (**a**) HepG2 cell proteins were extracted and PARP-1 and caspase-3 protein abundance was assessed by western blotting using specific antibodies. ß-actin was used as loading control. Graphs represent the quantification of cleaved PARP-1 and cleaved caspase-3 abundance normalized by the corresponding ß-actin in three independent experiences. Results are expressed as mean ± 1 S.D. (*n* = 3). (**b**) After the incubation with etoposide, caspase-3 and-7 activity was assayed in HepG2 cells by measuring the fluorescence intensity of free AFC released from the cleavage of Ac-DEVD-AFC. Results are expressed in relative caspase-3/-7 activity as mean ± 1 S.D. (*n* = 3). Statistical analysis was carried out with the one-way ANOVA test followed by a Holm-Sidak post-test. NS: no significantly different from control cells incubated with etoposide; * or **: significantly different from control cells incubated with etoposide respectively with p < 0.05 or 0.01

At the same time, protein extraction was performed on the HepG2 cells in order to measure apoptosis and how it could be affected by the co-cultured macrophages via secreted factors. Western blotting analyses were performed in order to measure cleaved caspase-3 and cleaved PARP-1 protein abundance (Fig. 9a) and caspase-3/7 activity was quantified using a fluorogenic substrate (Fig. 9b). An increased abundance of cleaved caspase-3 was observed in HepG2 cells incubated in the presence of M1 macrophages in comparison to control cancer cells incubated without macrophages. The slight increase in PARP-1 protein abundance was however not significant. M1 macrophages also increased the caspase activity in etoposide-exposed HepG2 cells. It has to be noted that a slight increase in HepG2 cell apoptosis was observed when cells were incubated with M1 macrophages in the absence of etoposide (data not shown). When HepG2 cells were incubated with M2 macrophages, cancer cell apoptosis was highly reduced in comparison to the one measured in control cells. Indeed, cleaved caspase-3 and cleaved PARP-1 proteins are much less abundant in cells incubated in the presence of M2 macrophages. Western blot results were confirmed by a caspase activity assay.

**Fig. 10:**
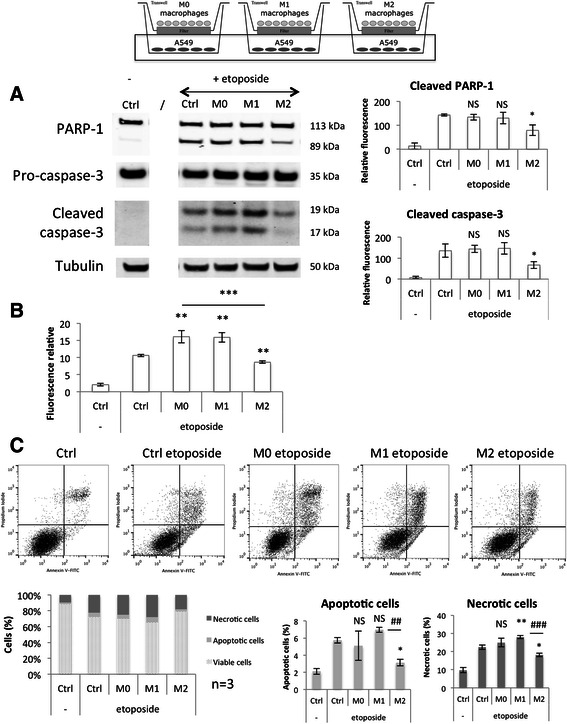
Modulation of A549 cell apoptosis induced by etoposide by co-cultured M0, M1 and M2 macrophages. Macrophages were co-cultured in indirect contact with A549 cells during 24 h before incubation with or without 50 μM etoposide (+/− e) during 16 h. (**a**) A549 cell proteins were extracted and PARP-1 and caspase-3 protein abundance was assessed by western blotting using specific antibodies. α-tubulin was used as loading control. Graphs represent the quantification of cleaved PARP-1 and cleaved caspase-3 abundance normalized by the corresponding α-tubulin in three independent experiences. Results are expressed as mean ± 1 S.D. (*n* = 3). (**b**) After the incubation with etoposide, caspase-3 and-7 activity was assayed in A549 cells by measuring the fluorescence intensity of free AFC released from the cleavage of Ac-DEVD-AFC. Results are expressed in relative caspase-3/-7 activity as mean ± 1 S.D. (*n* = 3). (**c**) After the incubation with macrophages, A549 cells were detached and stained with Annexin V-FITC and propidium iodide before fluorescence analysis by flow cytometry. The percentage of cells in the four different quadrants was calculated and the results present in different histograms where viable cells are Annexin V-/PI-, apoptotic cells Annexin V+/PI- and necrotic cells are PI +. Results are present as mean ± 1 S.D. (*n* = 3). Statistical analysis was carried out with the one-way ANOVA test followed by a Holm-Sidak post-test. NS: no significantly different from control cells incubated with etoposide; *, ** or ***: significantly different from control cells incubated with etoposide respectively with p < 0.05, 0.01 or 0.001

These results were reproduced in a second cancer cell line, A549 cells, co-cultured with macrophages during 24 h before addition of the etoposide and incubation during 16 h (Fig. 10). The incubation kinetic was changed because A549 cells are more sensitive to etoposide-induced apoptosis than HepG2 cells. M0 macrophages had no effect on the etoposide-induced HepG2 cell apoptosis (Fig. 9) and no effect on the etoposide-induced A549 cell apoptosis as measured by caspase-3 and PARP-1 cleavage (Fig. 10a) and propidium iodine-annexin V-labeling (Fig. 10c). However, an increase was observed for caspase-3/7 activity analysis in A549 cells (Fig. 10b), which is indeed no really consistent with the two other observations. This may be due to the activity of other caspases than caspase-3 like caspase-7.

In co-culture with A549 cells, the cytotoxic effect of M1 macrophages was smaller than what was observed in co-culture with HepG2 cells. However, M1 macrophages significantly increased the caspase activity as well as the percentage of propidium iodide positive A549 cells (necrotic cells) in comparison to control cells incubated with etoposide without macrophages (Fig. 10c). M1 macrophages increased the etoposide-induced HepG2 cell apoptosis (Fig. 9) as well as the etoposide-induced A549 cell apoptosis as measured by caspase-3 activity (Fig. 10b) and propidium iodine-annexin V-labeling (Fig. 10c). However, no significant effect was observed on cleaved caspase-3 and cleaved PARP-1 protein abundance in A549 cells (Fig. 10a), which is indeed no really consistent with the two other observations. However, for two western blot analyses out of the three independent experiments, this effect was observable.

On the opposite, M2 macrophages displayed a strong protective effect for all three parameters.

All together, these results showed that M1 and M2 macrophages differentiated and polarized from THP-1 monocytes modulate the apoptotic response to etoposide of two cancer cell lines, HepG2 and A549 cancer cells. M1 macrophages had a cytotoxic effect and increased the etoposide-induced apoptosis. On the opposite, M2 macrophages were protective and decreased the apoptosis in cancer cells exposed to this drug.

Using this *in vitro* model of co-culture, we were able to reproduce the effects of macrophages observed in clinical studies or with different *in vivo* animal models. Many studies have shown on one hand an inverse correlation between macrophage abundance in a tumor and patient prognosis and survival, and on the other hand a positive correlation with resistance to chemotherapy [11, 27–29]. In sites of chronic inflammation where a tumor may develop, macrophages have a M1 phenotype [30]. M1 macrophages are cytotoxic for pathogens and tumor cells. Their tumoricidal activity was related to their ability to secrete reactive nitrogen and oxygen species and pro-inflammatory cytokines [7]. THP-1 M1 macrophages polarized after 24 h incubation with 10 pg/ml of LPS and 20 ng/ml IFN-γ and incubated with HepG2 or A549 cells were able to increase the apoptosis of cancer cells induced by etoposide.

In malignant tumors, macrophages exhibit predominantly an M2-like phenotype [3, 31, 32]. M2 macrophages improve tumor cell growth and survival by secretion of many growth factors like EGF, members of the FGF family, TGF-ß or VEGF (vascular endothelial growth factor) [10, 33]. Many soluble factors present in the tumor microenvironment and secreted by macrophages have already been described to decrease cancer cell response to chemotherapy like IL-1ß [34, 35], VEGF [36], TGF-ß [37], IL-4 [38] as well as cathepsins B and S [11]. However, not all of these genes are typical M2 macrophage markers. Indeed, IL-1ß is up regulated in M1 macrophages and cathepsin S is slightly up regulated after IFN-γ and LPS stimulation (data not shown).

THP-1 M2 macrophages polarized by 72 h incubation with 20 ng/ml of IL-4 and IL-13 and incubated with HepG2 or A549 cells highly reduced the etoposide-induced apoptosis. It has to be noted that, in human macrophages, NO production is not modulated by polarization as it is described for murine macrophages [5]. This is important since etoposide has been shown to be chemically modified by NO-derived species and forms products with reduced toxic activity [39].

Etoposide at the concentration used in this work did not influence macrophage polarization. Moreover macrophage expression profiles were very similar between co-culture and monoculture experiments performed in the same incubation conditions. It means that cancer cells have no impact on THP-1 macrophage polarization after incubation with etoposide. Results from Weigert *et al*. [40] showed that when primary macrophages were incubated in direct co-culture with MCF-7 cells, they produced TNF-α that induced MCF-7 cell apoptosis. Apoptotic cells released sphingosine-1-phosphate (S1P), which caused a macrophage phenotypic switch from M1 to M2. THP-1 M0 macrophages did not induce cancer cell apoptosis in our co-culture system and no phenotypic switch was observed after co-culture with etoposide. However, the kinetic used in that study was more than 3 days longer than ours. The same research group also described that S1P can favor macrophage survival after etoposide incubation [39]. We did not study the impact of S1P on THP-1 macrophage survival, which seemed unaffected by 24 h incubation with 50 μM of etoposide, but we did it on HepG2 cell response to etoposide. S1P had no effect on etoposide-induced apoptosis of HepG2 cells (data not shown). Hence, the differential effects we observed were not due to a shift from one phenotype to the other.

Our model is original because it uses THP-1 differentiated macrophages, which are easy to obtain, differentiate and polarize. M1 and M2 THP-1 macrophages have the same expression profiles than polarized primary macrophages. We studied the influence of macrophages on cancer cell response to etoposide. Macrophage pre-incubation with cancer cells was needed to obtain a protective effect of M2 macrophages on the etoposide-induced apoptosis. It means that the two cell populations have to exchange soluble factors, which will activate pro-survival pathways allowing cancer cells to resist to the etoposide-induced apoptosis. This model is a great tool to study the influence of a specific pathway in the protective or cytotoxic effect of macrophages on cancer cells. We used it to study the influence of the COX-I pathway (up-regulated in the M2 macrophages, data not shown) and no effect of COX-I inhibition was observed on the protective effect of M2 macrophages.

## Conclusion

In conclusion, we developed an easy, reproducible and well-characterized model of differentiated THP-1 monocytes polarized into M1 and M2 macrophages. Using this model, we demonstrated that M1 and M2 macrophages exerted opposite effects on cancer cell response to a chemotherapeutic drug.

## Abbreviations

BSA: Bovine serum albumin
CD206 (=MRC1): Mannose receptor
IRDye: Infrared dye
PARP1: Poly [ADP-ribose] polymerase 1
PMA: Phorbol 12-myristate 13-acetate
RPS9: 40S ribosomal protein S9
TAM: Tumor associated macrophage
